# Identifying α-KG-dependent prognostic signature for lower-grade glioma based on transcriptome profiles

**DOI:** 10.3389/fonc.2022.840394

**Published:** 2022-07-27

**Authors:** Tan Zhang, Liqun Yuan, Minfeng Sheng, Yanming Chen, Ji Wang, Qing Lan

**Affiliations:** Department of Neurosurgery, The Second Affiliated Hospital of Soochow University, Suzhou, China

**Keywords:** IDH mutation, α-KG, 2-HG, lower-grade glioma, genome-wide analysis

## Abstract

The inhibition of alpha-ketoglutarate (α-KG)-dependent dioxygenases is thought to contribute to isocitrate dehydrogenase (*IDH*) mutation-derived malignancy. Herein, we aim to thoroughly investigate the expression pattern and prognostic significance of genes encoding α-KG-dependent enzymes for lower-grade glioma (LGG) patients. In this retrospective study, a total of 775 LGG patients were enrolled. The generalized linear model, least absolute shrinkage and selection operator Cox regression, and nomogram were applied to identify the enzyme-based signature. With the use of gene set enrichment analysis and Gene Ontology, the probable molecular abnormalities underlying high-risk patients were investigated. By comprehensively analyzing mRNA data, we observed that 41 genes were differentially expressed between IDH^MUT^ and IDH^WT^ LGG patients. A risk signature comprising 10 genes, which could divide samples into high- and low-risk groups of distinct prognoses, was developed and independently validated. This enzyme-based signature was indicative of a more malignant phenotype. The nomogram model incorporating the risk signature, molecular biomarkers, and clinicopathological parameters proved the incremental utility of the α-KG-dependent signature by achieving a more accurate prediction impact. Our study demonstrates that the α-KG-dependent enzyme-encoding genes were differentially expressed in relation to the *IDH* phenotype and may serve as a promising indicator for clinical outcomes of LGG patients.

## Introduction

Lower-grade gliomas (LGGs; World Health Organization [WHO] grades 2 and 3) are diffuse brain tumors that are designated as astrocytoma and oligodendroglioma on the basis of histopathological and molecular criteria (1). The highly infiltrative and biologically heterogeneous nature of LGG frequently results in tumor recurrence or malignant progression (2). According to the Chinese Glioma Genome Atlas (CGGA) statistics, the median overall survival (OS) times are 78.1 months for WHO grade 2 gliomas and only 37.6 months for anaplastic gliomas (WHO grade 3) (3). Notably, clinical results with LGG range from a few months to more than 10 years, and some patients exhibit remarkable treatment sensitivity (4). This variation illustrates the need for integrative research to explain the molecular processes behind LGG malignancy.

A genome-wide analysis recently identified mutations of the isocitrate dehydrogenase (*IDH*) genes in 70%–90% of LGGs (5). IDH mutations impart a neomorphic enzyme activity that catalyzes the reduction of alpha-ketoglutarate (α-KG) into the suspected oncometabolite 2-hydroxyglutarate (2-HG), resulting in an accumulation of 2-HG in IDH mutant gliomas (6, 7). Accumulating investigations have revealed that the *IDH* mutations served as critical indicators for glioma diagnosis, classification, and prognostic prediction (5). Although the mutations of *IDH* appear to cause widespread disruptions in cellular physiology, particularly the epigenome (6), the molecular mechanisms by which *IDH* mutations promote gliomagenesis remain to be clarified.

α-KG is an intermediary in the tricarboxylic acid cycle and a crucial cosubstrate for over 60 dioxygenases, including JmjC domain-containing enzymes, DNA/RNA-modifying enzymes, proline/lysine hydroxylases, and other hydroxylases (8). 2-HG is structurally identical to α-KG, with the exception that 2-HG has a C2 hydroxyl group instead of a C2 carbonyl (6). Exogenous expression of tumor-derived IDH mutants suppresses histone demethylation and 5-methylcytosine hydroxylation, supporting the hypothesis that 2-HG may act as a competitive inhibitor of α-KG-dependent enzymes that control a variety of physiological activities (9). Exploration of the roles and prognostic implications of α-KG-dependent enzymes will thereby increase our understanding of the genetic abnormalities associated with IDH mutation. In the present study, transcriptome data from CGGA were utilized for screening *IDH*-related α-KG-dependent genes. These genes were then subjected to the least absolute shrinkage and selection operator (Lasso) Cox regression model to select the most useful prognostic features. With the coefficients generated by Lasso Cox, an enzyme-based risk evaluating model was identified and independently validated. The high-risk score was indicative of a worse outcome and a malignant phenotype for LGG patients. Our findings indicated that α-KG-dependent enzymes may be utilized as a robust tool for prognostication, which has tremendous promise in glioma-specific treatment.

## Materials and methods

### Patients and transcriptome data

Whole transcriptome sequencing data and corresponding clinical information (histology, gender, age, grade, therapeutic approaches, and survival information) were downloaded from the CGGA database (http://www.cgga.org.cn) as the training set. *IDH* mutations were detected using pyrosequencing and performed on a Pyro-Mark Q96 ID System (Qiagen, Valencia, CA, USA) as previously described (10). Primers used for PCR amplification are listed as follows: IDH1 5′-GCTTGTGAGTGGATGGGTAAAAC-3′ and 5′-Biotin-TTGCCAACATGACTTACTTGATC-3′; IDH2 5′-ATCCTGGGGGGGACTGTCTT-3′ and 5′-Biotin-CTCTCCACCCTGGCCTACCT-3′. The primer sequences used for pyrosequencing are 5′-TGGATGGGTAAAACCT-3′ for IDH1 and 5′-AGCCCATCACCATTG-3′ for IDH2. The status of chromosome 1p/19q was calculated as reported (11). A total of 362 LGG patients with clinical characteristics, transcriptome sequencing, and molecular data (ATRX, 1p/19q, IDH) were downloaded from The Cancer Genome Atlas (TCGA) as the validation set (http://cancergenome.nih.gov/). Whole-genome mRNA expression microarray data and clinical information from GSE16011 and Repository of Molecular Brain Neoplasia Data (Rembrandt) were obtained for analyses as well. Moreover, the Genotype-Tissue Expression (GTEx) transcriptome data from GEPIA2 was obtained to compare the expression between normal and tumor tissues.

### Integration of genes encoding α-KG-dependent dioxygenases

The known and putative α-KG-dependent dioxygenases were obtained as previously reported, including 10 DNA/RNA-modifying enzymes (*TET1*, *TET2*, *TET3*, *ABH1*, *ABH2*, *ABH3*, *ABH4*, *ABH5*, *ABH6*, and *FTO*), 29 JmjC domain-containing enzymes (*KDM2A*, *KDM2B*, *KDM3A*, *KDM3B*, *KDM4A*, *KDM4B*, *KDM4C*, *KDM4D*, *KDM5A*, *KDM5B*, *KDM5C*, *KDM5D*, *KDM6A*, *KDM6B*, *KDM7A*, *KDM8*, *HR*, *JARID2*, *JHDM1C*, *JMJD1C*, *JMJD4*, *JMJD6*, *JMJD7*, *JMJD8*, *MINA*, *NO66*, *PHF2*, *PHF8*, and *UTY*), 15 proline/lysine hydroxylases (*EGLN1*, *EGLN2*, *EGLN3*, *P4HA1*, *P4HA2*, *P4HA3*, *P4HB*, *P4HTM*, *PLOD1*, *PLOD2*, *PLOD3*, *LEPRE1*, *LEPREL1*, *LEPREL2*, and *TMLHE*), and 11 other hydroxylases (*ASPH*, *ASPHD1*, *ASPHD2*, *BBOX1*, *FIH1*, *HSPBAP1*, *OGFOD1*, *OGFOD2*, *PAHX-AP1*, *PHYH*, and *PHYHD1*) (12).

### Identification of *IDH*-related genes

To identify differential genes between *IDH* wild type (IDH^WT^) and *IDH* mutant (IDH^MUT^) patients, Student’s *t*-test was applied, and genes with corresponding *p*< 0.05 were considered as candidates. The diagnostic performance of the *IDH*-related gene signature was measured using generalized linear models (GLMs) embedded in Matlab software. The GLM generalizes linear regression by allowing the linear model to be linked to the response variable and by allowing the magnitude of the variance of each measurement to be a function of its predicted value. A receiver operating characteristic (ROC) curve with the calculated area under the curve (AUC) was delineated based on the screened genes.

### Construction and validation of the enzyme-based risk score

According to Harrell’s guideline, the number of samples should exceed at least 10 times of included variables in a multivariate analysis. To address this issue, we performed the Lasso Cox regression model for dimensionality reduction (13). The selected genes were used in developing a linear combination weighted by their respective coefficients generated by the Lasso Cox model. The risk score for the OS time of each individual was calculated as follows:


$$Risk\;score\;=\;expr_{gene1}\times \;C_{gene1}+\;expr_{gene2}\times \;C_{gene2}+\cdots +expr_{gene10}\times C_{gene10}$$

Next, the patients in the training dataset were classified into high-risk and low-risk groups using the median risk score as the cutoff point. The same coefficients for each gene and median risk score cutoff were applied to TCGA, GSE16011, and Rembrandt datasets for validation.

### Gene ontology and gene set enrichment analysis

To classify the latent molecular alterations between high- and low-risk patients, we screened the genes significantly associated with the evaluation of risk scores. The gene with Pearson’s correlation coefficient greater than 0.5 and a *p*-value less than 0.05 were subjected to Gene Ontology (GO) analysis using the online Database for Annotation, Visualization and Integrated Discovery (DAVID; http://david.ncifcrf.gov/) (14). Heatmaps were constructed using Gene Cluster and Gene Tree View software. Top significant biological processes or cellular components were figured with R programming language (http://cran.r-project.org). Meanwhile, we explored the differences between risk groups utilizing gene set enrichment analysis (GSEA). The annotated gene sets were obtained from the Molecular Signatures Database v5.1 (MSigDB) (http://www.broad.mit.edu/gsea/msigdb/).

### Pharmacogenomic interaction analysis

To identify therapeutic approaches that can be used to specifically treat LGG patients with a worse risk score, genetic and pharmacological profiles were obtained from the Catalogue of Somatic Mutations in Cancer (COSMIC, http://cancer.sanger.ac.uk/cell_lines) (15). Pearson’s correlation algorithm was utilized to identify the correlation between the IC_50_ value of anti-cancer drugs and gene expression data.

### Statistical analysis

The significant differences between the two groups were estimated using Student’s *t*-test. The chi-square test and Fisher’s exact test were used to compare the frequencies between groups. The Kaplan–Meier survival analysis was implemented to estimate the survival distributions. Cox regression was used to determine the prognostic value of each variable with OS in LGG patients. Nomograms were depicted based on the results of a backward step-down selection process with the Akaike information criterion (AIC) by using the package of *rms* in R (13, 16). The performance of the nomogram was measured by the concordance index (C-index), which was indicative of the accuracy of the prognostic prediction (16). Calibration curves were drawn for each dataset for predicted 1-, 2-, and 3-year survival. All differences were considered statistically significant at the level of two-sided *p*< 0.05. Statistics were carried out using the SPSS 13.0 statistical software and GraphPad Prism 6.0.

## Results

### Identification of *IDH*-associated enzyme genes

**Figure 1:**
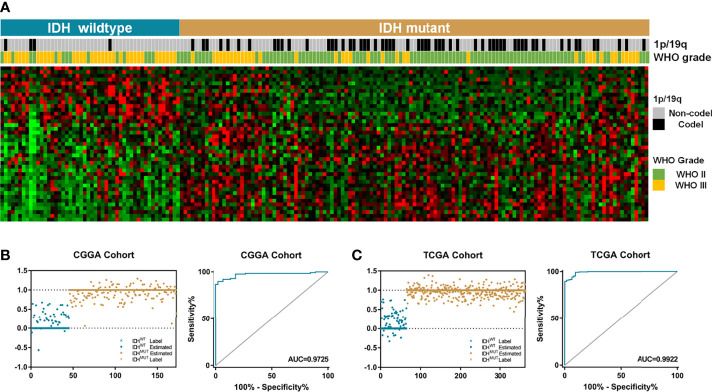
The IDH-associated α-KG-dependent gene signature. **(A)** The LGG patients were classified into two groups based on relevant IDH status. A total of 41 genes were differentially expressed between IDH^WT^ and IDH^MUT^ groups. **(B)** The signature comprising 41 genes could be used to distinguish IDH phenotypes with an AUC of 0.973 in CGGA dataset. **(C)** In TCGA cohort, the enzyme-based signature could divide LGG samples into two groups with an AUC of 0.992. α-KG, alpha-ketoglutarate; LGG, lower-grade glioma; AUC, area under the curve; CGGA, Chinese Glioma Genome Atlas; TCGA, The Cancer Genome Atlas.

To evaluate the relationship between *IDH* mutation and enzyme-encoding genes in LGG patients, we first screened the differentially expressed genes between the IDH^WT^ and IDH^MUT^ groups. A total of 65 known and putative α-KG-dependent dioxygenases were utilized for analyses (12). After genes with no significance were eliminated, an enzyme-related signature of 41 genes was established (**Figure 1A**). Among which, 26 were upregulated and 15 were downregulated in IDH^MUT^ LGGs compared to the IDH^WT^ counterpart. Then, we applied the GLM algorithm to access the diagnostic value of these genes in discriminating IDH phenotype. In the CGGA cohort, these 41 genes could distinguish LGG patients as IDH^WT^ and IDH^MUT^ with an AUC of 0.973 (**Figure 1B**), while this signature could stratify samples with an AUC of 0.992 in TCGA cohort (**Figure 1C**).

### Construction of a 10-gene-based risk evaluation formula

**Figure 2:**
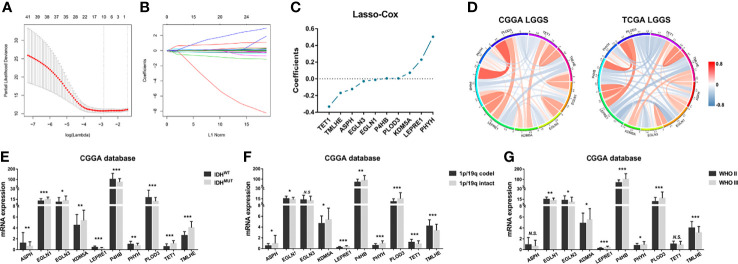
Construction of enzyme-based prognostic signature. **(A, B)** The 10-fold cross-validation for Lasso Cox analysis identified 10 genes with a non-zero coefficient. **(C)** The genes with non-zero coefficients in Lasso were plotted. **(D)** These 10 genes showed similar relationship in both CGGA and TCGA datasets. **(E)** The expression patterns of these genes were compared between IDH^MUT^ and IDH^WT^ groups, **(F, G)** 1p/19q intact and codeletion groups, as well as WHO II and WHO III groups. *p< 0.05, **p< 0.01, and ***p< 0.001. Lasso, least absolute shrinkage and selection operator; CGGA, Chinese Glioma Genome Atlas; TCGA, The Cancer Genome Atlas; NS, No Significance.

**Figure 3:**
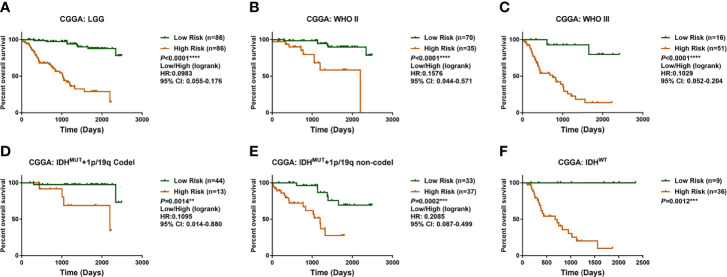
Identification and validation of prognostic significance for the enzyme-based risk signature. **(A)** LGG patients with high-risk scores survived shorter than the low-risk patients in CGGA cohort. The enzyme-based risk signature could be used to stratify patients into two populations with distinct overall survival times regarding WHO grade **(B, C)** and LGG subgroups **(D–F)**. **p< 0.01, ***p< 0.001 and ****p< 0.0001. LGG, lower-grade glioma; CGGA, Chinese Glioma Genome Atlas.

Previous studies highlight the importance of *IDH* mutation in prognostic prediction for LGG patients. To determine the prognostic significance of these differential genes, the Lasso Cox algorithm was used to pick the most potent prognostic characteristics from the CGGA dataset (**Figures 2A**, **B**). The genes with a non-zero coefficient in the Lasso Cox regression model were *TET1* (−0.418), *TMLHE* (−0.179), *ASPH* (−0.142), *EGLN3* (−0.031), *EGLN1* (−0.013), *PLOD3* (0.004), *P4HB* (0.006), *KDM5A* (0.080), *LEPRE1* (0.235), and *PHYH* (0.559) (**Figure 2C**). These 10 genes presented a similar correlation in both the CGGA and TCGA cohorts (**Figure 2D**) and were significantly dysregulated between different IDH phenotypes, WHO grades, and chromosome 1p/19q status (**Figures 2E-G**). *EGLN1*, *KDM5A*, *P4HB*, and *PLOD3* were statistically significant between normal and LGG tissues (**Figure S1**). Moreover, these genes could serve as predictors for OS of LGG patients (**Figures S2**, **S3**). To access the general efficiency and effectiveness of the 10 genes comprised signature in prognostic prediction, we calculated the risk score of each individual based on the gene expression levels and corresponding regression coefficients. Patients were classified into a high-risk group (n = 86) and a low-risk group (n = 86) based on the median risk value as the cutoff. Notably, patients with lower risk score survived longer than those in the high-risk group (hazard ratio [HR] = 0.0983, 95% confidence interval [CI]: 0.055–0.176, *p*< 0.0001; **Figure 3A**). Furthermore, this signature-based risk score could divide WHO II and III patients into two groups with the distinct OS using the same cutoff (**Figures 3B, C**). Similarly, in different subtypes of LGG, this signature could be used for the prediction of prognosis (**Figures 3D-F**). Cox regression analysis revealed that this risk model was associated with the survival of patients, independent of the clinical parameters (HR = 2.642, 95% CI: 1.612–4.332, *p*< 0.001; **Table 1**).

**Table 1:** Univariate and multivariate Cox analyses in CGGA and TCGA LGG samples.

	Univariate			Multivariate		
	HR	95% CI	*p*	HR	95% CI	*p*
***CGGA datasets***						
**Age**	1.049	1.020–1.078	**0.001**			
**Sex**						
Male *vs*. female	1.078	0.608–1.908	0.798			
**WHO grade**						
2 *vs*. 3	0.166	0.090–0.306	**<0.001**	0.334	0.161–0.696	**0.003**
**IDH status**						
Mutant *vs*. wild type	0.252	0.142–0.445	**<0.001**			
**1p/19q status**						
Codeletion *vs*. non-codeletion	0.188	0.084–0.420	**<0.001**	0.375	0.152–0.925	**0.033**
**Radiotherapy**						
Yes *vs*. no	0.536	0.276–1.040	0.065			
**Risk score**	4.256	3.044–5.951	**<0.001**	2.642	1.612–4.332	**<0.001**
***TCGA datasets***						
** Age**	1.067	1.047–1.087	**<0.001**	1.066	1.046–1.086	**<0.001**
** Sex**						
Female *vs*. male	0.995	0.628–1.578	0.983			
**WHO grade**						
2 *vs*. 3	0.290	0.172–0.490	**<0.001**	0.489	0.281–0.849	**0.011**
**IDH status**						
Mutant *vs*. wild type	0.172	0.106–0.277	**<0.001**	0.342	0.189–0.619	**<0.001**
**1p/19q status**						
Codeletion *vs*. non-codeletion	0.424	0.237–0.758	**0.004**			
**Risk score**	2.336	1.672–0.268	**<0.001**	1.667	1.148–2.415	**0.007**

HR, hazard ratio; 95% CI, 95% confidence interval; CGGA, Chinese Glioma Genome Atlas; TCGA, The Cancer Genome Atlas; LGG, lower-grade glioma. Bold values have statistically significant.

### Validation of the risk signature

The prognostic value of the α-KG-dependent enzymes was validated using TCGA RNA sequencing data. The same formula was applied to calculate the risk score of each sample in the validating datasets, and the median risk score was used as a grouping criterion. In TCGA cohort, the survival curve showed that LGG patients with high-risk scores had a shorter OS than low-risk ones (*p*< 0.0001; **Figure S4**). Cox regression analysis revealed that the signature-based risk score could serve as an independent predictive indicator for LGG patients (*p* = 0.007; **Table 1**). To further confirm the robustness of this risk signature, microarray data from GSE16011 and Rembrandt datasets were analyzed. Consistently, the OS of LGG patients in GSE16011 and Rembrandt dataset could be stratified distinctly (**Figures S4**, **S5**), while the multivariate Cox model revealed that this risk signature could serve as an independent indicator for LGG patients (GSE16011, HR = 1.965, 95% CI: 1.475–2.618, *p*< 0.001; Rembrandt, HR = 2.890, 95% CI: 1.789–4.672, *p*< 0.001; **Table S1**).

### High risk indicated a malignant phenotype of lower-grade glioma

**Figure 4:**
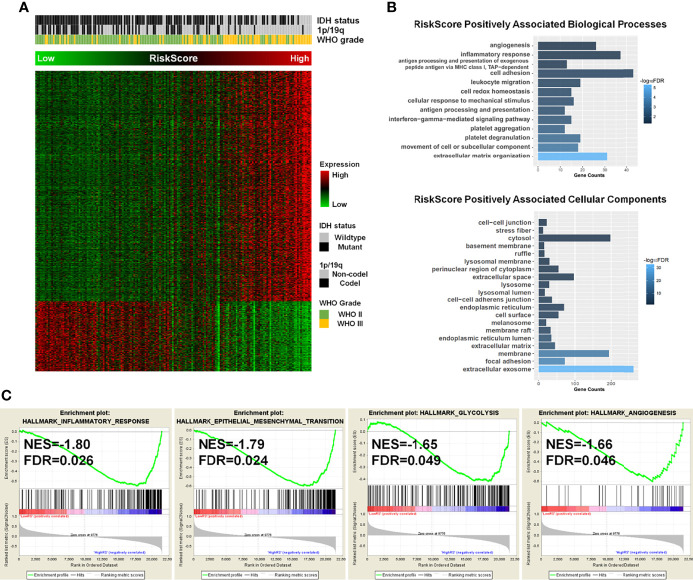
The potential molecular alterations of high-risk LGG patients. **(A)** Associations between the enzyme-based risk value and the clinicopathological features and its related genes. **(B)** Gene Ontology analysis of biological processes and cellular components for high-risk positively associated genes. **(C)** Gene set enrichment analysis revealed that the hallmark gene sets of cancer, including inflammatory response, epithelial–mesenchymal transition, glycolysis, and angiogenesis, were significantly enriched in high-risk groups. LGG, lower-grade glioma.

Considering the significance of the risk score in prognostic prediction, we attempted to explore the latent molecular alterations. GO analysis of the high-risk positively related genes revealed that several malignant biological processes, including angiogenesis, cell adhesion, inflammatory response, and extracellular matrix organization, were significantly enriched (**Figures 4A, B**). Similarly, the GO results of TCGA, GSE16011, and Rembrandt suggested that the biological processes were mainly in relation to cell adhesion, angiogenesis, inflammatory response, apoptotic process, and cell proliferation (**Figure S6**). Afterward, the differences in clinical and pathological characteristics between high- and low-risk populations were compared. There are more WHO grade 3 gliomas, lower IDH mutation frequency, and lower 1p/19q codeletion ratio in the high-risk group in both the CGGA and TCGA datasets (**Table 2**). In GSE16011 sets, the low-risk group showed a higher IDH mutation frequency, while high-risk patients in Rembrandt sets were diagnosed with more WHO grade 3 glioma (**Figure S7**). GSEA identified that significant hallmarks involved in the high-risk group were inflammatory response, epithelial–mesenchymal transition, glycolysis, and angiogenesis (**Figure 4C**).

**Table 2:** Clinical characteristics of LGG patients in CGGA and TCGA datasets.

	CGGA dataset				TCGA dataset		
	Low risk (n = 86)	High risk (n = 86)	*p*		Low risk (n = 181)	High risk (n = 181)	*p*
**Age (mean)**	39.3	41.5	0.199^a^		42.4	44.0	0.236^a^
**Sex**							
Female	34	33	>0.999^b^		82	85	0.833^b^
Male	52	53			99	96	
**WHO grade**							
WHO 2	70	35	**<0.001** ^b^		101	71	**0.002** ^b^
WHO 3	16	51			80	110	
**WHO 2016**							
A, IDH^MUT^	17	24	**<0.001** ^b^		62	82	**<0.001** ^b^
A, IDH^WT^	3	22			0	49	
O, IDH^WT^, codeletion	43	11			103	22	
O, NOS	8	3			16	28	
**IDH**							
Mutant	77	50	**<0.001** ^b^		181	117	**<0.001** ^b^
Wild type	9	36			0	64	
**1p/19q**							
Codeletion	45	16	**<0.001** ^b^		103	22	**<0.001** ^b^
Non-codeletion	41	70			78	159	

A, astrocytoma; O, oligodendroglioma; LGG, lower-grade glioma; CGGA, Chinese Glioma Genome Atlas; TCGA, The Cancer Genome Atlas.

a t-Test.

b Fisher’s exact test or chi-square. Bold values have statistically significant.

### Assessment of enzyme-based risk signature in lower-grade glioma overall survival performance

**Figure 5:**
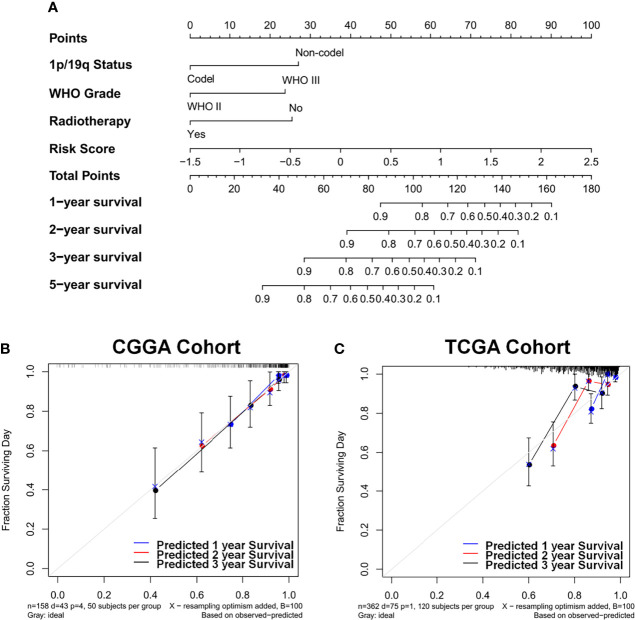
Identification and construction of the nomogram to estimate the overall survival for LGG patients. **(A)** The constructed nomogram estimated the risk of patients’ prognosis for 1, 2, 3, and 5 years in CGGA cohort. Calibration curves showed the calibration of each model in terms of the agreement between the estimated and observed 1-year (blue), 2-year (red), and 3-year (black) outcomes in CGGA **(B)** and TCGA **(C)** datasets. Diagonal line presents a perfect estimation of an ideal model, while colored lines stand for the performance of the nomogram. A closer distance to the diagonal line represents a better estimation. LGG, lower-grade glioma; CGGA, Chinese Glioma Genome Atlas; TCGA, The Cancer Genome Atlas.

The prognostic nomogram that integrated all significant factors derived from AIC selection was depicted. In the CGGA cohort, the integrated nomogram with an enzyme-based risk signature could increase the C-index from 0.803 to 0.856 (**Figure 5A**). The calibration curves for the probability of survival at 1, 2, or 3 years showed an optimal agreement between the estimation and actual observation (**Figure 5B**). Moreover, this nomogram was independently validated in TCGA cohort. The C-index for validating the nomogram is 0.754, which showed a good calibration as well (**Figure 5C**).

### High-risk lower-grade glioma patients may benefit from PI3K/Akt targeted therapy

To explore the therapeutic strategies for the high-risk population, the pharmacogenomic interaction dates of LGG cell lines were analyzed. With the exacerbation of risk score, LGG cell lines presented a higher IC_50_ value for a spectrum of anti-cancer drugs, such as tozasertib, paclitaxel, and sorafenib. On the contrary, PI3K/Akt targeted drugs, including pictilisib (PI3K), MK-2206 (Akt1, Akt2), NU7441 (DNAPK, PI3K), AZD6482 (PI3K), and idelalisib (PI3K), effectively inhibited cell viability *in vitro*, which is attributed to the hyperactivation of PI3K/Akt signaling pathway in high-risk tumors (**Figure S8**). Interestingly, the IC_50_ of JQ-1, a BET bromodomain inhibitor, was negatively correlated with the enzyme-based risk score (**Figure S8D**), suggesting a therapeutic implication for epigenetic inhibition in the treatment of this population.

## Discussion

IDH mutations have altered the molecular categorization and prognostication of glioma patients through somatic mutations. The abnormal accumulation of 2-HG in IDH mutant gliomas and the structural similarities between 2-HG and α-KG provide evidence for the concept that 2-HG might serve as an α-KG antagonist to influence many cellular processes by hydroxylating target proteins utilizing α-KG as a cosubstrate (6, 17). In the present study, we aimed to explore the transcriptomic alterations and prognostic value of α-KG-dependent dioxygenases between IDH^WT^ and IDH^MUT^ LGG samples. By comprehensively analyzing whole-genome mRNA expression data from training and discovery datasets, we demonstrated that 41 enzyme-based genes with high sensitivity and specificity could differentiate between various IDH phenotypes. Survival analyses further identified a risk signature comprising 10 genes, which showed powerful capability in prognostic prediction and indicated a more malignant phenotype for LGG patients. Notably, integrating the enzyme-based risk signature, molecular biomarkers, and clinicopathological features into a nomogram had a superior predictive impact, demonstrating the added utility of the α-KG-dependent prognostic signature for LGG-specific medication.

IDH mutation-derived 2-HG was proved to competitively inhibit the histone demethylases and TET family of 5-methylcytosine hydroxylases, leading to increased methylation of histone H3 and decreased 5-hydroxymethylcytosine (18–20). Therefore, it is plausible to hypothesize that the changed expression and activity of α-KG-dependent dioxygenases may contribute to the IDH mutation-mediated phenotypic alteration. Their significance to categorization and prognosis, however, has yet to be demonstrated. Here, we demonstrated that 41 α-KG-dependent dioxygenase-encoding genes can function as surrogate indicators for IDH mutation. Among them, EGLN1 and EGLN3, two α-KG-dependent dioxygenases of the EglN prolyl-4-hydroxylase family (21), were upregulated in IDH mutant LGG patients and served as favorable indicators for LGG prognosis, which is consistent with previous studies proving that 2-HG specifically increased the activity of EglN enzymes in human astrocytes (22) and that overexpression of EGLN3 could suppress tumor progression of glioma models by normalized glioma capillary architecture and tightly associated with hypoxic environment (23, 24). As the main component of the extracellular matrix, collagen can modify tumor cell behavior and promote tumor development (25). Here, we revealed that POLD3 and P4HB, two genes involved in collagen metabolism, were significantly dysregulated according to IDH status, which is consistent with previous studies (26, 27). TET enzymes are another family of α-KG-dependent enzymes, which are thought to play important roles in the epigenetic regulation of gene expression. Although studies have proved that TET enzyme activity was decreased by ectopic addition of 2-HG (20), the expression pattern of TET mRNAs has not been well investigated. Our results showed that TET1 was upregulated in IDH mutant samples, and the lower expression of TET1 indicated a worse prognosis for LGG patients. Similar consequences were observed by former investigations that low levels of TET1 were associated with reduced survival in glioblastoma (28). These enzymes appear to have multiple functions and show great potential as novel therapeutic targets (12, 29, 30). Further investigations are needed to investigate the functional relationship between IDH mutation and other dioxygenase genes, such as KDM5A, a histone demethylase and a key factor for the resistance to temozolomide in glioblastoma (31).

The genetic analyses revealed that the high-risk score was associated with several malignant biological processes, including angiogenesis, cell adhesion, and inflammatory response. These hallmarks constituted a more malignant phenotype of cancer (32), which effectively deciphered the relationship between high-risk patients and worse clinical outcomes. Previous studies demonstrated that IDH mutations are indicative of a favorable prognosis, whereas patients with equal IDH status always exhibit distinct outcomes (17, 33). Importantly, our results revealed that the enzyme-based risk signature was shown to have prognostic significance for IDH mutant LGG; thereby, we propose that our signature is a useful supplement for the development of IDH^MUT^ individual management. We also suggested that the risk signature was significantly associated with angiogenesis, hypoxia, proliferation, and several oncogenic pathways, including JAK/STAT3, PI3K/Akt/mTOR, and TNFα/NFκB (**Figure S7**). Recognition of these concepts will increasingly affect the development of new approaches to treat glioma by targeting the α-KG-dependent dioxygenases and thus improving the clinical outcome.

Nomograms are widely used because of their ability to reduce statistical predictive models into a single numerical estimate of the probability of an event that is tailored to the profile of an individual patient (34). The combination of prognostic molecular signatures and clinical risk factors has shown enhanced prognostic accuracy. An established nomogram integrating multiple risk factors, including age at diagnosis, gender, resection, Karnofsky Performance Status, and MGMT promoter methylation, successfully estimated the OS for patients with resected GBM (C-index: 0.657) (35). Our study, for the first time, demonstrated that the combined clinical, pathological, and molecular nomogram achieved better prognostic performance and calibration with a higher C-index in LGG patients. Meanwhile, the nomogram model was externally validated in TCGA cohort, confirming the incremental value of this enzyme-based risk signature in individualized estimation. For widespread clinical application, future assessments in a prospective longitudinal cohort with more comprehensive prognostic features should be performed.

## Conclusion

Collectively, the developed and confirmed enzyme-based signature, which was predictive of a more malignant phenotype for LGG patients, has the potential to act as a surrogate biomarker and prognostic indicator for IDH phenotype and clinical outcome. The risk signature-based nomogram has the potential to help in the formulation of an effective therapeutic strategy and the direction of postoperative care for patients with glioma.

## Data availability statement

The datasets presented in this study can be found in online repositories. The names of the repository/repositories and accession number(s) can be found in the article/**Supplementary Material**.

## Author contributions

TZ: method design, writing—original draft, and writing—review and editing. LY: writing—original draft. MS: data analysis and sorting. YC: writing—review and editing. JW: revised the manuscript. QL: conceptualization, method design, and writing editing. All authors contributed to the article and approved the submitted version.

## Conflict of interest

The authors declare that the research was conducted in the absence of any commercial or financial relationships that could be construed as a potential conflict of interest.

## Publisher’s note

All claims expressed in this article are solely those of the authors and do not necessarily represent those of their affiliated organizations, or those of the publisher, the editors and the reviewers. Any product that may be evaluated in this article, or claim that may be made by its manufacturer, is not guaranteed or endorsed by the publisher.

## Supplementary material

Supplementary Figure 1 The expression of candidate genes between normal and lower-grade gliomas in GTEx and TCGA.

Supplementary Figure 2 Survival analyses for individual genes in CGGA cohorts.

Supplementary Figure 3 Survival analyses for individual genes in TCGA cohorts.

Supplementary Figure 4 Prognostic significance of the enzyme-based risk signature in TCGA and Rembrandt datasets.

Supplementary Figure 5 Prognostic significance of the enzyme-based risk signature in GSE16011 dataset.

Supplementary Figure 6 Biological Processes and cellular components analyse of risk-score positively associated genes in TCGA, GSE16011 and Rembrandt datasets.

Supplementary Figure 7 Associations between the risk value and the clinicopathologic features in GSE16011 and Rembrandt cohorts.

Supplementary Figure 8 Pharmacogenomic interaction analysis. **(A)** Heatmap of targeted drugs with R>0.35 or R<-0.35. **(B-G)** Pearson correlation analyses of candidate drugs.

## References

[1] Louis DN Perry A Reifenberger G von Deimling A Figarella-Branger D Cavenee WK. The 2016 world health organization classification of tumors of the central nervous system: a summary. Acta Neuropathol. (2016) 131:803–20. doi: 10.1007/s00401-016-1545-1 27157931

[2] Gittleman H Sloan AE Barnholtz-Sloan JS. An independently validated survival nomogram for lower-grade glioma. Neuro Oncol (2020) 22:665–74. doi: 10.1093/neuonc/noz191 PMC7229246 31621885

[3] Jiang T Mao Y Ma W Mao Q You Y Yang X. CGCG clinical practice guidelines for the management of adult diffuse gliomas. Cancer Lett (2016) 375:263–73. doi: 10.1016/j.canlet.2016.01.024 26966000

[4] Brat DJ Verhaak RG Aldape KD Yung WK Salama SR Cooper LA. Comprehensive, integrative genomic analysis of diffuse lower-grade gliomas. N Engl J Med (2015) 372:2481–98. doi: 10.1056/NEJMoa1402121 PMC4530011 26061751

[5] Pirozzi CJ Yan H. The implications of IDH mutations for cancer development and therapy. Nat Rev Clin Oncol (2021) 18:645–61. doi: 10.1038/s41571-021-00521-0 34131315

[6] Waitkus MS Diplas BH Yan H. Isocitrate dehydrogenase mutations in gliomas. Neuro Oncol (2016) 18:16–26. doi: 10.1093/neuonc/nov136 26188014 PMC4677412

[7] Kadiyala P Carney SV Gauss JC Garcia-Fabiani MB Haase S Alghamri MS. Inhibition of 2-hydroxyglutarate elicits metabolic reprogramming and mutant IDH1 glioma immunity in mice. J Clin Invest. (2021) 131:e139542. doi: 10.1172/JCI139542 PMC7880418 33332283

[8] Baksh SC Finley L. Metabolic coordination of cell fate by α-ketoglutarate-dependent dioxygenases. Trends Cell Biol (2021) 31:24–36. doi: 10.1016/j.tcb.2020.09.010 33092942 PMC7748998

[9] Waitkus MS Diplas BH Yan H. Biological role and therapeutic potential of idh mutations in cancer. Cancer Cell (2018) 34:186–95. doi: 10.1016/j.ccell.2018.04.011 PMC6092238 29805076

[10] Cai J Zhang W Yang P Wang Y Li M Zhang C. Identification of a 6-cytokine prognostic signature in patients with primary glioblastoma harboring M2 microglia/macrophage phenotype relevance. PLoS One (2015) 10:e0126022. doi: 10.1371/journal.pone.0126022 25978454 PMC4433225

[11] Hu X Martinez-Ledesma E Zheng S Kim H Barthel F Jiang T. Multigene signature for predicting prognosis of patients with 1p19q co-deletion diffuse glioma. Neuro Oncol (2017) 19:786–95. doi: 10.1093/neuonc/now285 PMC5464432 28340142

[12] Losman JA Kaelin WG. What a difference a hydroxyl makes: mutant IDH, (R)-2-hydroxyglutarate, and cancer. Genes Dev (2013) 27:836–52. doi: 10.1101/gad.217406.113 PMC3650222 23630074

[13] Huang Y Liu Z He L Chen X Pan D Ma Z. Radiomics signature: A potential biomarker for the prediction of disease-free survival in early-stage (I or II) non-small cell lung cancer. Radiology (2016) 281:947–57. doi: 10.1148/radiol.2016152234 27347764

[14] Huang da W Sherman BT Lempicki RA. Systematic and integrative analysis of large gene lists using DAVID bioinformatics resources. Nat Protoc (2009) 4:44–57. doi: 10.1038/nprot.2008.211 19131956

[15] Forbes SA Beare D Boutselakis H Bamford S Bindal N Tate J. COSMIC: somatic cancer genetics at high-resolution. Nucleic Acids Res (2017) 45:D777–777D783. doi: 10.1093/nar/gkw1121 27899578 PMC5210583

[16] Wang Y Li J Xia Y Gong R Wang K Yan Z. Prognostic nomogram for intrahepatic cholangiocarcinoma after partial hepatectomy. J Clin Oncol (2013) 31:1188–95. doi: 10.1200/JCO.2012.41.5984 23358969

[17] Yan H Parsons DW Jin G McLendon R Rasheed BA Yuan W. IDH1 and IDH2 mutations in gliomas. N Engl J Med (2009) 360:765–73. doi: 10.1056/NEJMoa0808710 PMC2820383 19228619

[18] Chowdhury R Yeoh KK Tian YM Hillringhaus L Bagg EA Rose NR. The oncometabolite 2-hydroxyglutarate inhibits histone lysine demethylases. EMBO Rep (2011) 12:463–9. doi: 10.1038/embor.2011.43 PMC3090014 21460794

[19] Lu C Ward PS Kapoor GS Rohle D Turcan S Abdel-Wahab O. IDH mutation impairs histone demethylation and results in a block to cell differentiation. Nature (2012) 483:474–8. doi: 10.1038/nature10860 PMC3478770 22343901

[20] Xu W Yang H Liu Y Yang Y Wang P Kim SH. Oncometabolite 2-hydroxyglutarate is a competitive inhibitor of α-ketoglutarate-dependent dioxygenases. Cancer Cell (2011) 19:17–30. doi: 10.1016/j.ccr.2010.12.014 21251613 PMC3229304

[21] Villar D Vara-Vega A Landázuri MO Del Peso L. Identification of a region on hypoxia-inducible-factor prolyl 4-hydroxylases that determines their specificity for the oxygen degradation domains. Biochem J (2007) 408:231–40. doi: 10.1042/BJ20071052 PMC2267344 17725546

[22] Koivunen P Lee S Duncan CG Lopez G Lu G Ramkissoon S. Transformation by the (R)-enantiomer of 2-hydroxyglutarate linked to EGLN activation. Nature (2012) 483:484–8. doi: 10.1038/nature10898 PMC3656605 22343896

[23] Sciorra VA Sanchez MA Kunibe A Wurmser AE. Suppression of glioma progression by Egln3. PLoS One (2012) 7:e40053. doi: 10.1371/journal.pone.0040053 22905089 PMC3414484

[24] Strocchi S Reggiani F Gobbi G Ciarrocchi A Sancisi V. The multifaceted role of EGLN family prolyl hydroxylases in cancer: going beyond HIF regulation. Oncogene (2022). doi: 10.1038/s41388-022-02378-8 35705735

[25] Shi R Gao S Zhang J Xu J Graham LM Yang X. Collagen prolyl 4-hydroxylases modify tumor progression. Acta Biochim Biophys Sin (Shanghai). (2021) 53:805–14. doi: 10.1093/abbs/gmab065 PMC8445723 34009234

[26] Tsai CK Huang LC Tsai WC Huang SM Lee JT Hueng DY. Overexpression of PLOD3 promotes tumor progression and poor prognosis in gliomas. Oncotarget (2018) 9:15705–20. doi: 10.18632/oncotarget.2459 PMC5884658 29644003

[27] Zou H Wen C Peng Z YΥ S Hu L Li S. P4HB and PDIA3 are associated with tumor progression and therapeutic outcome of diffuse gliomas. Oncol Rep (2018) 39:501–10. doi: 10.3892/or.2017.6134 PMC5783617 29207176

[28] Orr BA Haffner MC Nelson WG Yegnasubramanian S Eberhart CG. Decreased 5-hydroxymethylcytosine is associated with neural progenitor phenotype in normal brain and shorter survival in malignant glioma. PLoS One (2012) 7:e41036. doi: 10.1371/journal.pone.0041036 22829908 PMC3400598

[29] Forristal CE Winkler IG Nowlan B Barbier V Walkinshaw G Levesque JP. Pharmacologic stabilization of HIF-1α increases hematopoietic stem cell quiescence *in vivo* and accelerates blood recovery after severe irradiation. Blood (2013) 121:759–69. doi: 10.1182/blood-2012-02-408419 23243286

[30] Myllyharju J. HIF prolyl 4-hydroxylases and their potential as drug targets. Curr Pharm Des (2009) 15:3878–85. doi: 10.2174/138161209789649457 19671043

[31] Banelli B Carra E Barbieri F Würth R Parodi F Pattarozzi A. The histone demethylase KDM5A is a key factor for the resistance to temozolomide in glioblastoma. Cell Cycle (2015) 14:3418–29. doi: 10.1080/15384101.2015.1090063 PMC4825557 26566863

[32] Mitchell K Troike K Silver DJ Lathia JD. The evolution of the cancer stem cell state in glioblastoma: emerging insights into the next generation of functional interactions. Neuro Oncol (2021) 23:199–213. doi: 10.1093/neuonc/noaa259 33173943 PMC7906055

[33] Zhang CB Bao ZS Wang HJ Yan W Liu YW Li MY. Correlation of IDH1/2 mutation with clinicopathologic factors and prognosis in anaplastic gliomas: a report of 203 patients from China. J Cancer Res Clin Oncol (2014) 140:45–51. doi: 10.1007/s00432-013-1519-9 24149775 PMC11824052

[34] Iasonos A Schrag D Raj GV Panageas KS. How to build and interpret a nomogram for cancer prognosis. J Clin Oncol (2008) 26:1364–70. doi: 10.1200/JCO.2007.12.9791 18323559

[35] Gittleman H Lim D Kattan MW Chakravarti A Gilbert MR Lassman AB. An independently validated nomogram for individualized estimation of survival among patients with newly diagnosed glioblastoma: NRG oncology RTOG 0525 and 0825. Neuro Oncol (2016) 19:669–77. doi: 10.1093/neuonc/now208 PMC5464437 28453749

