# Supplementary material for: Identifying α-KG-dependent prognostic signature for lower-grade glioma based on transcriptome profiles
Source: Front Oncol. 2022 Jul 27;12:840394. doi: 10.3389/fonc.2022.840394 (PMC9363673; doi:10.3389/fonc.2022.840394)
Supplement: Supplementary file 1 [file Table_1.docx]

Supplementary Table 1. Multivariate cox analysis in GSE16011 and Rembrandt LGG samples

|  |  | **Multivariate** | | |
| --- | --- | --- | --- | --- |
|  |  | **HR** | **95% CI** | ***P*** |
| ***GSE16011 datasets*** |  |  |  |  |
| **Age** |  | 0.945 | 0.923-0.967 | **<0.001** |
| **Sex** |  |  |  |  |
| Female vs. Male |  | 0.830 | 0.474-1.451 | 0.513 |
| **WHO Grade** |  |  |  |  |
| 2 vs. 3 |  | 0.769 | 0.381-1.553 | 0.464 |
| **Histology type** |  |  |  |  |
| O/OA vs. A |  | 0.658 | 0.316-1.370 | 0.263 |
| **IDH Status** |  |  |  |  |
| Mutant vs. Wildtype |  | 1.636 | 0.866-3.090 | 0.129 |
| **KPS** |  |  |  |  |
| ≥80 vs. <80 |  | 0.949 | 0.488-1.849 | 0.879 |
| **Resection** |  |  |  |  |
| Complete vs. Other |  | 0.701 | 0.371-1.327 | 0.275 |
| **RiskScore** |  | 1.965 | 1.475-2.618 | **<0.001** |
| ***Rembrandt datasets*** |  |  |  |  |
| **Sex** |  |  |  |  |
| Female vs. Male |  | 1.095 | 0.656-1.828 | 0.728 |
| **WHO Grade** |  |  |  |  |
| 2 vs. 3 |  | 0.684 | 0.409-1.143 | 0.464 |
| **Histology type** |  |  |  |  |
| O/OA vs. A |  | 0.905 | 0.530-1.545 | 0.715 |
| **RiskScore** |  | 2.890 | 1.789-4.672 | **<0.001** |

A, astrocytoma; O, oligodendroglioma; OA, oligoastrocytoma

HR, hazard ratio; 95% CI, 95% confidence interval
