# Supplementary figures and images for: Identifying α-KG-dependent prognostic signature for lower-grade glioma based on transcriptome profiles
Source: Front Oncol. 2022 Jul 27;12:840394. doi: 10.3389/fonc.2022.840394 (PMC9363673; doi:10.3389/fonc.2022.840394)

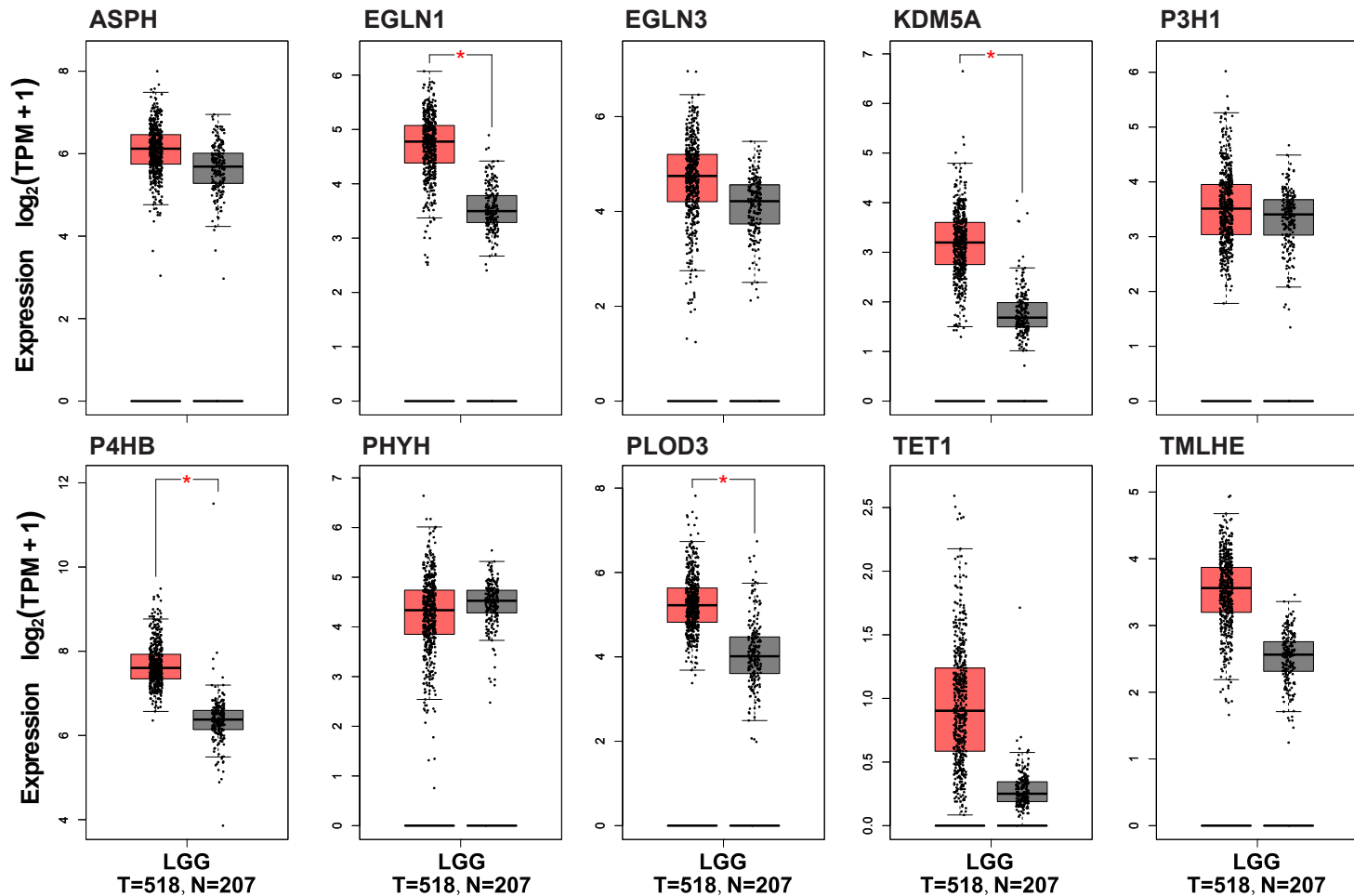

Supplement: Supplementary Figure 1 — The expression of candidate genes between normal and lower-grade gliomas in GTEx and TCGA. [file DataSheet_1.pdf]

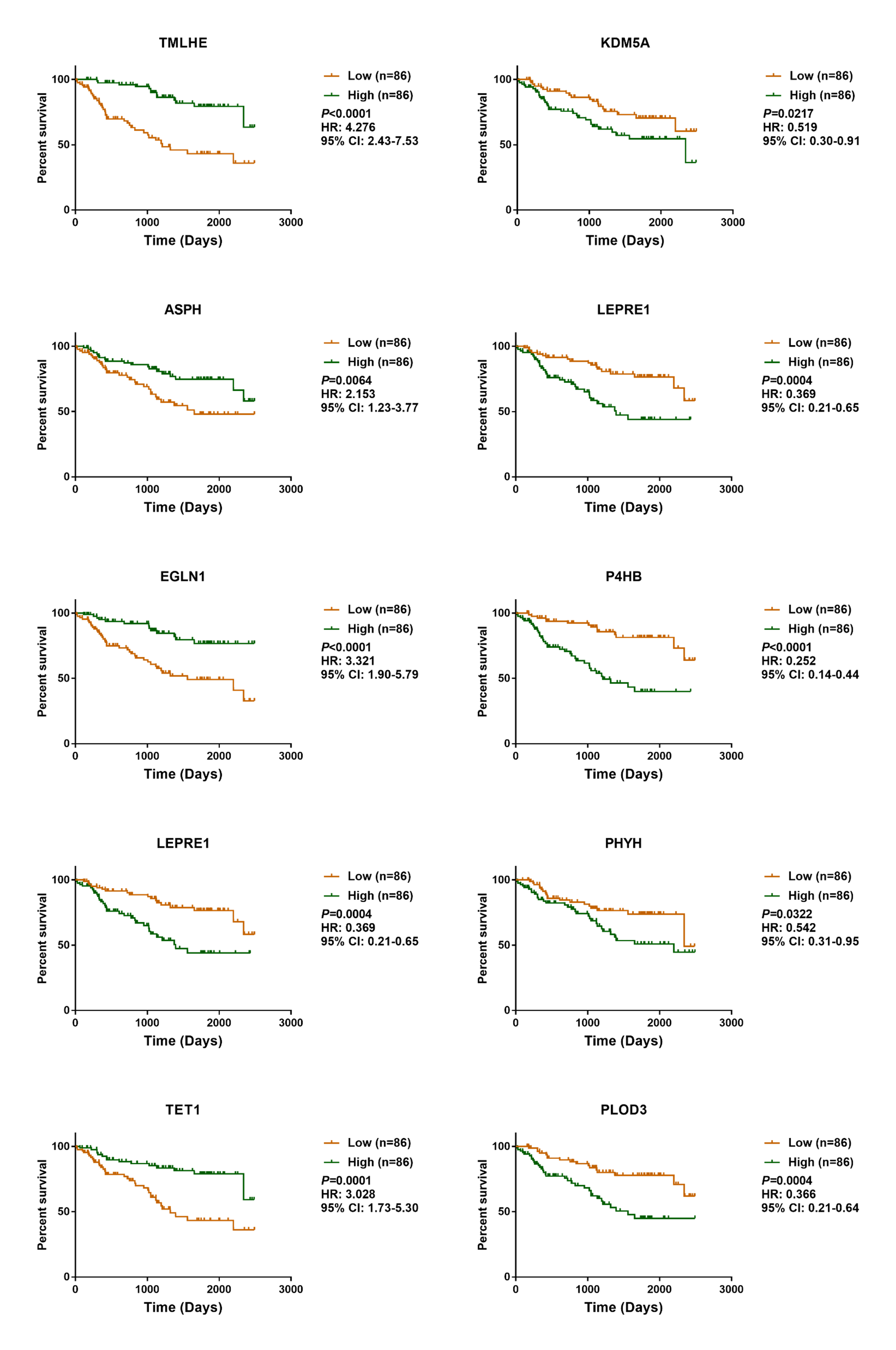

Supplement: Supplementary Figure 2 — Survival analyses for individual genes in CGGA cohorts. [file Image_1.tif]

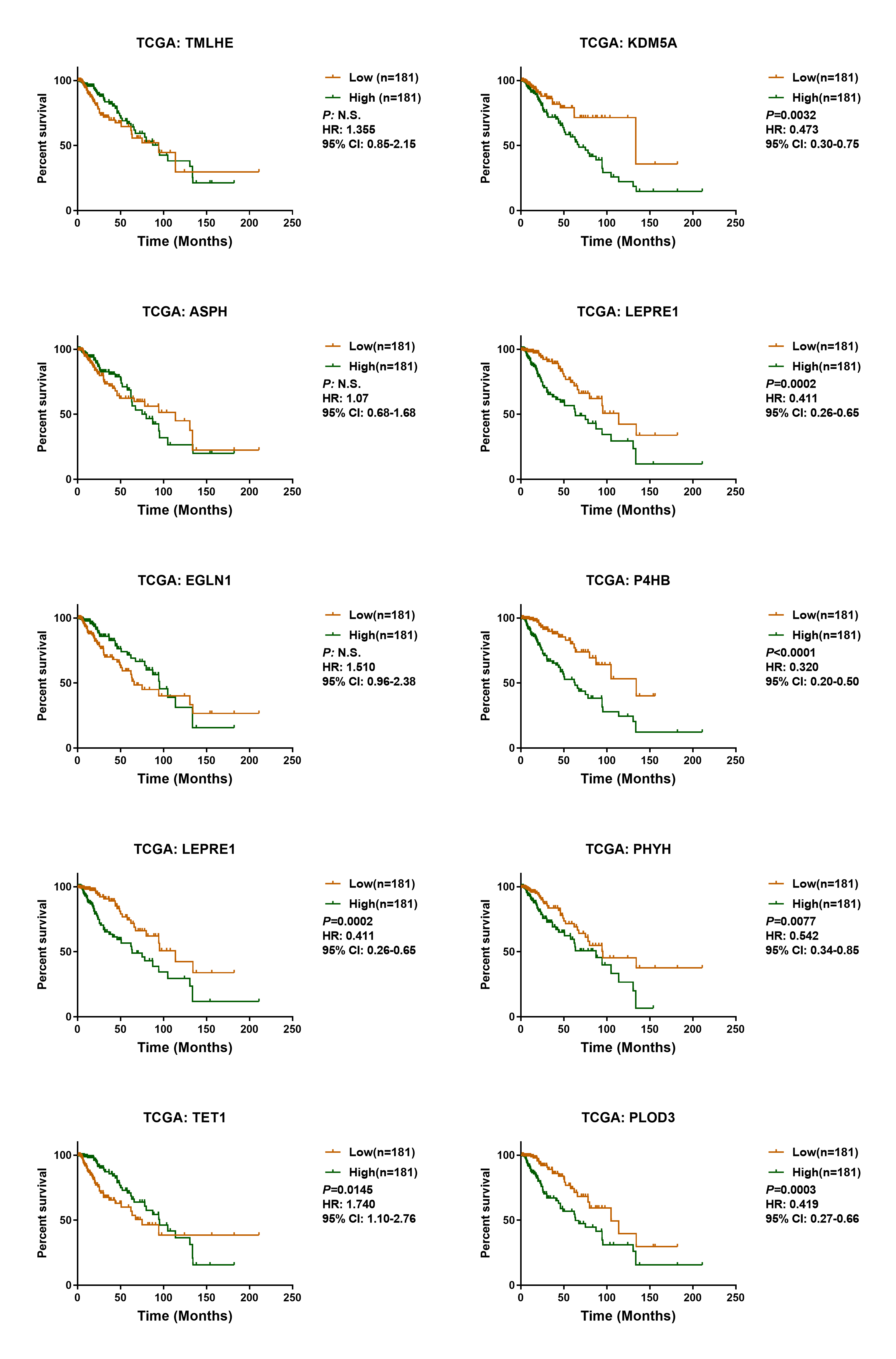

Supplement: Supplementary Figure 3 — Survival analyses for individual genes in TCGA cohorts. [file Image_2.tif]

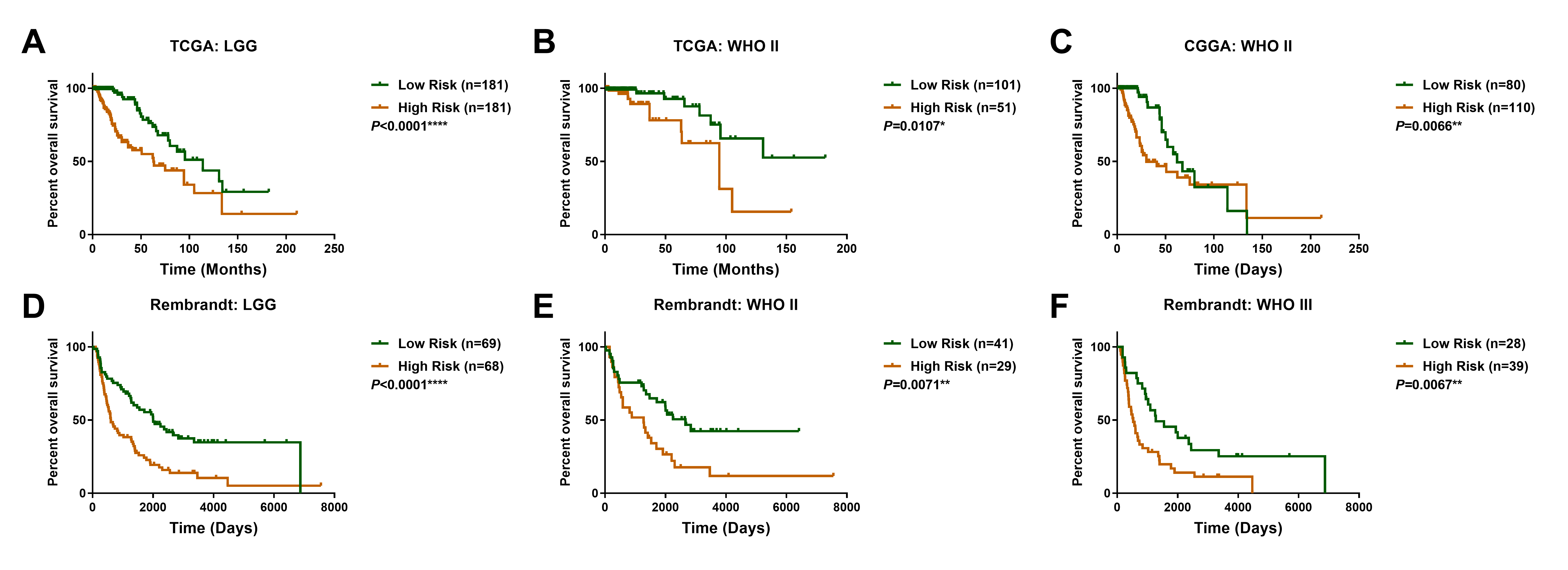

Supplement: Supplementary Figure 4 — Prognostic significance of the enzyme-based risk signature in TCGA and Rembrandt datasets. [file Image_3.tif]

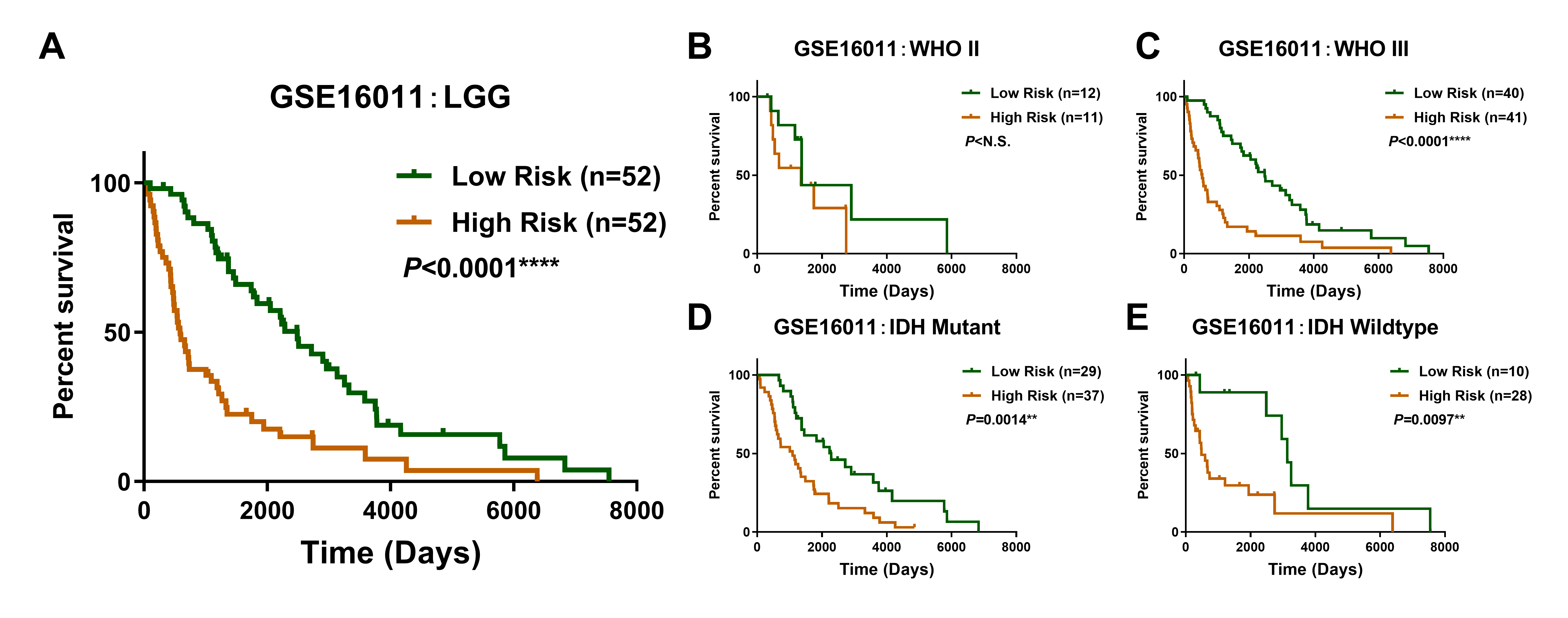

Supplement: Supplementary Figure 5 — Prognostic significance of the enzyme-based risk signature in GSE16011 dataset. [file Image_4.tif]

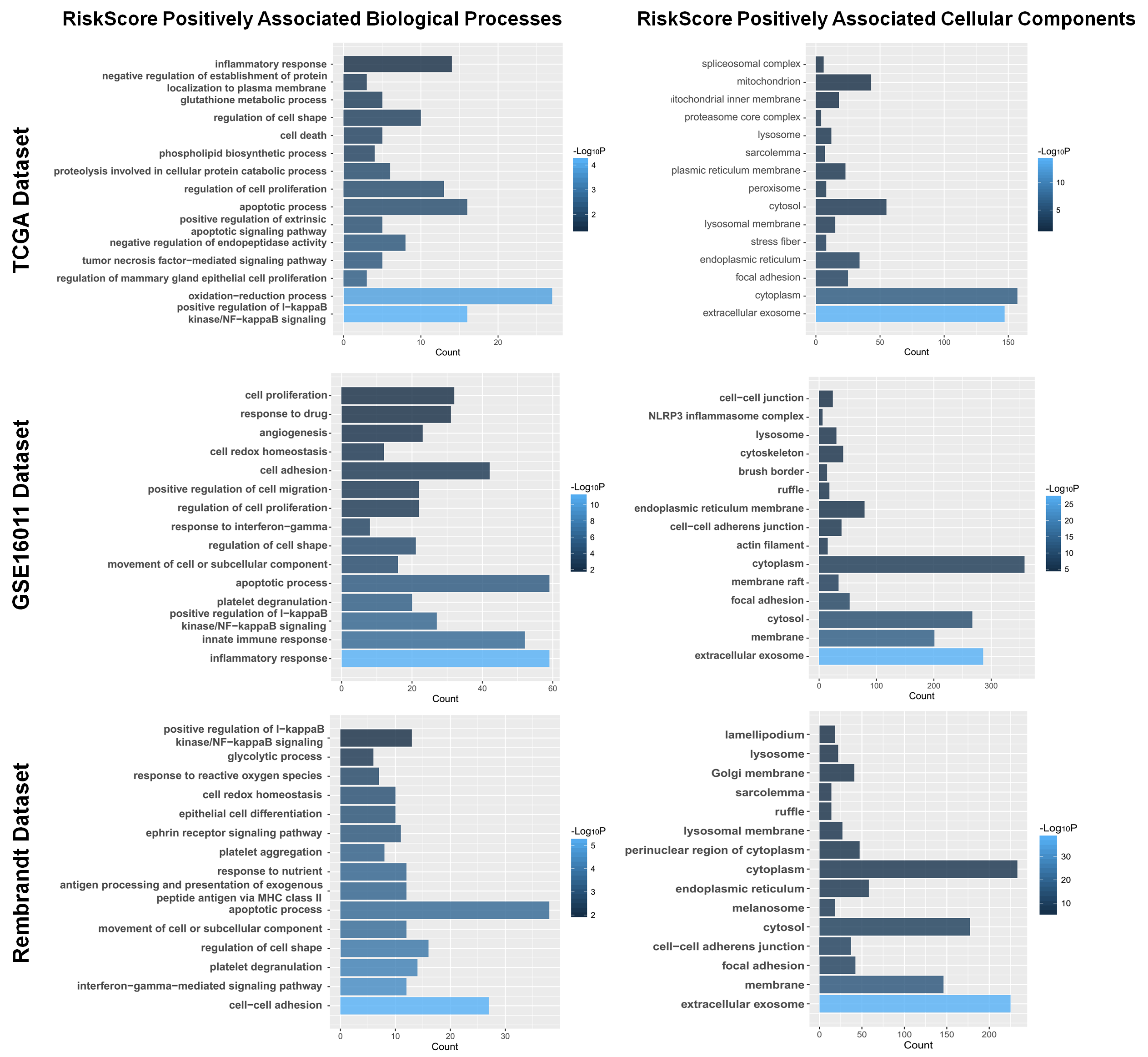

Supplement: Supplementary Figure 6 — Biological Processes and cellular components analyse of risk-score positively associated genes in TCGA, GSE16011 and Rembrandt datasets. [file Image_5.tif]

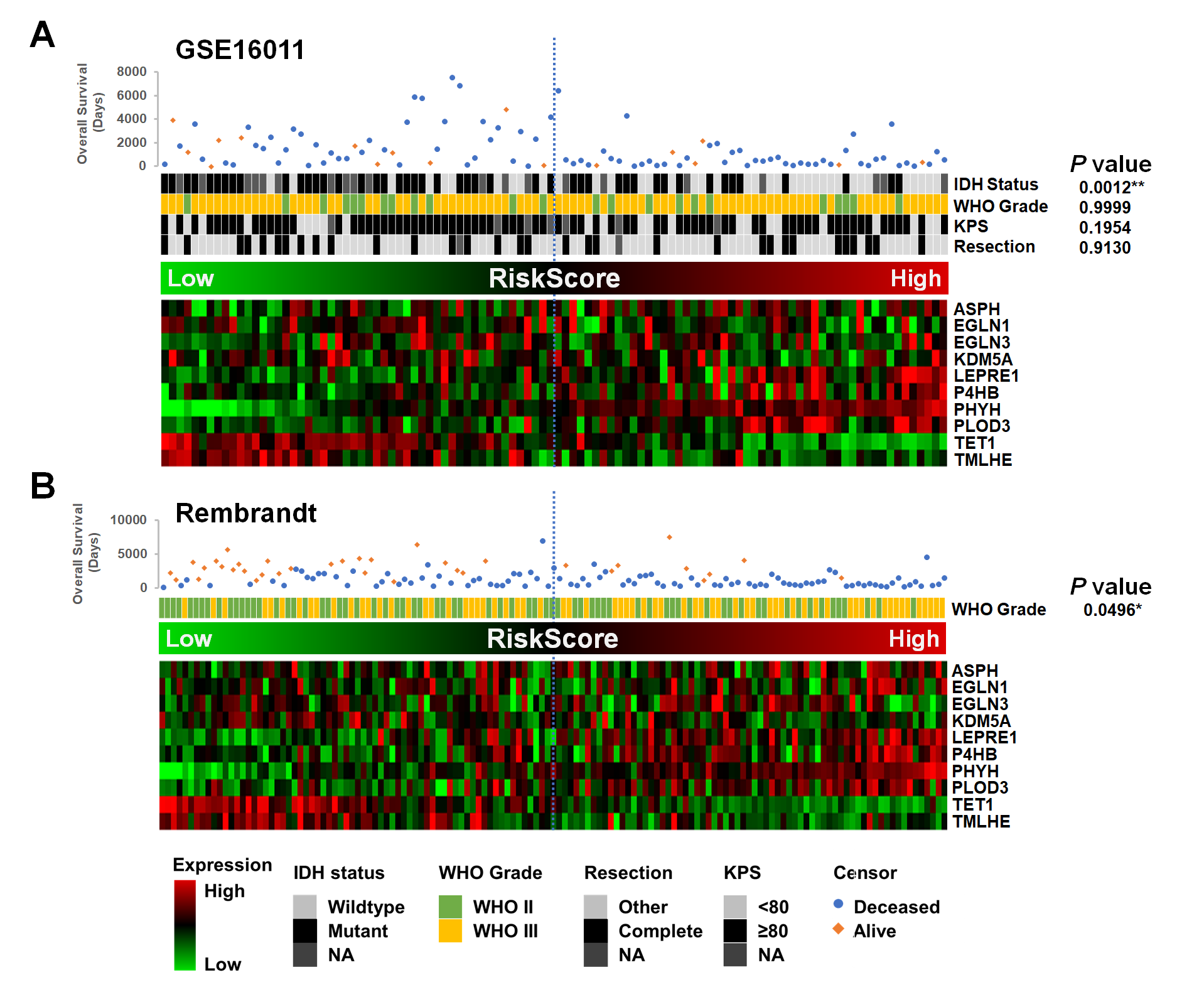

Supplement: Supplementary Figure 7 — Associations between the risk value and the clinicopathologic features in GSE16011 and Rembrandt cohorts. [file Image_6.tif]

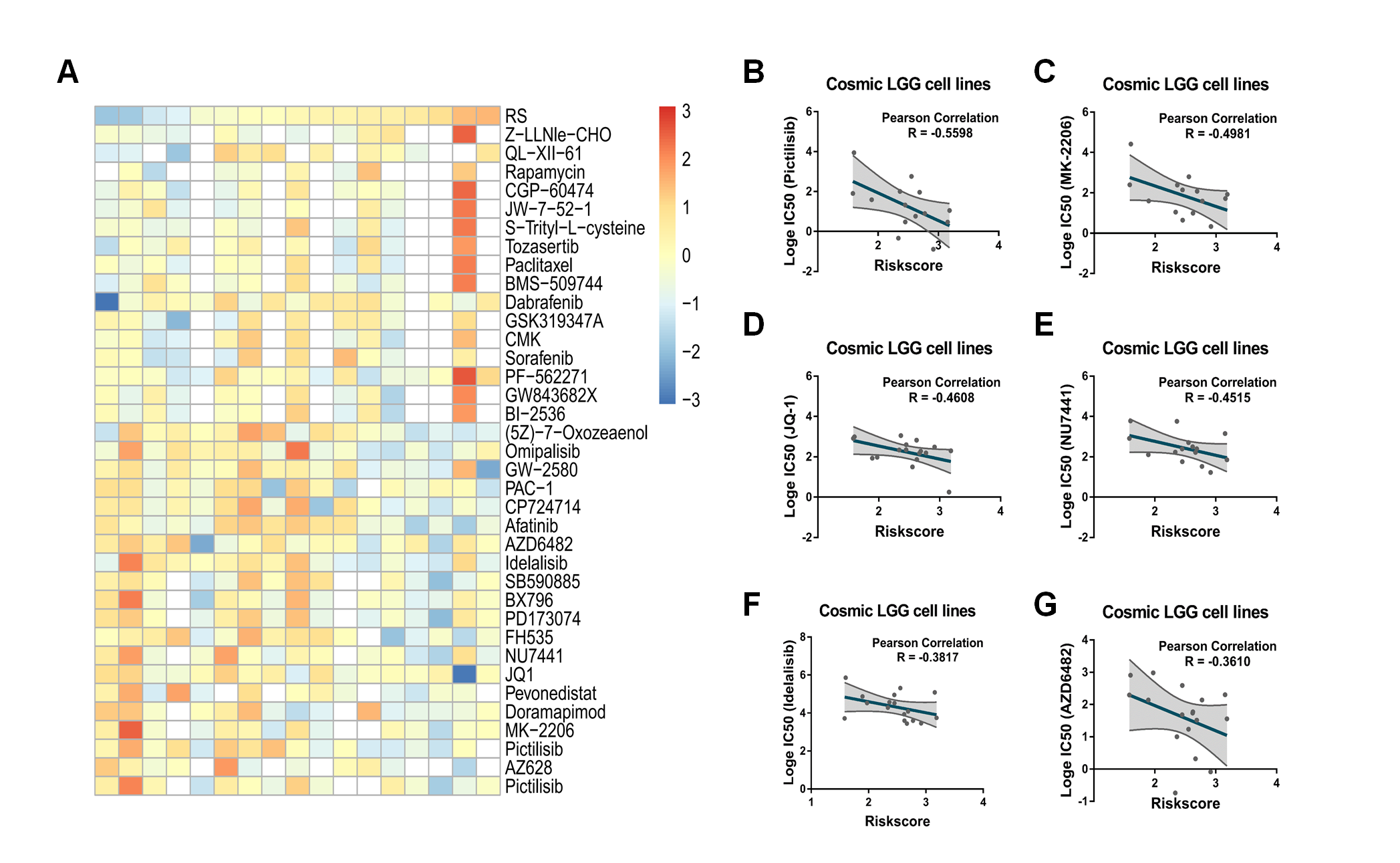

Supplement: Supplementary Figure 8 — Pharmacogenomic interaction analysis. (A) Heatmap of targeted drugs with R>0.35 or R<-0.35. (B-G) Pearson correlation analyses of candidate drugs. [file Image_7.tif]
